# Enrichment and Assessment of the Contributions of the Major Polyphenols to the Total Antioxidant Activity of Onion Extracts: A Fractionation by Flash Chromatography Approach

**DOI:** 10.3390/antiox7120175

**Published:** 2018-11-27

**Authors:** Mohammad B. Hossain, Justine Lebelle, Rares Birsan, Dilip K. Rai

**Affiliations:** TeagascFood Research Centre, Ashtown, Dublin D15KN3K, Ireland; Justine.lebelle@gmail.com (J.L.); Rares.Birsan@teagasc.ie (R.B.); Dilip.rai@teagasc.ie (D.K.R.)

**Keywords:** onion, antioxidant, polyphenols, fractionation, flash chromatography, LC-MS/MS

## Abstract

The present study extensively fractionated crude red onion extract in order to identify the polyphenols which contributed most in the total antioxidant capacity of the onion extract using a flash chromatography system. The flash separations produced 70 fractions which were tested for their total phenol content, total flavonoid content, and antioxidant capacities as measured by 2,2-diphenyl-1-picrylhydrazyl (DPPH) and ferric reducing antioxidant power (FRAP) assays. Out of these 70 fractions, four fractions which were representatives of the four major peaks of the flash chromatograms, were further analysed for their constituent polyphenols using liquid chromatography tandem mass spectrometry (LC-MS/MS). The main contributor of onion antioxidant capacity is quercetin glycoside followed by quercetin aglycone although quercetin aglycone had higher antioxidant capacity than its glycosidic counterparts. High abundance of quercetin glycosides such as quercetin-3,4′-diglucoside and quercetin-4′-glucoside had compensated for their relatively low antioxidant capacities. A Higher degree of glycosylation resulted in lower antioxidant capacity. The fractionation approach also contributed in enrichment of the onion antioxidant polyphenols. A >9 folds enrichment was possible by discarding the early fractions (fractions 1–15) which contained the main bulk of the extracts, predominantly sugars.

## 1. Introduction

Reactive oxygen species of both endogenous and exogenous origin are present inside a biological system. These chemicals cause oxidation of cellular molecules and lead to development of degenerative diseases such as cancer, diabetes, arthritis, cardio-vascular diseases, and neurodegenerative diseases through immunomodulation [1]. In food, the oxidation is largely responsible for the loss or development of undesired colour, texture, flavour, and nutritional quality in foods [2]. It is for this reason research interests focus on natural antioxidants in foods with a view to minimizing damages caused by free radicals and maintaining food quality [1,2,3,4,5,6,7,8]. 

Consumption of diet rich in vegetables and fruits has been associated with reduced risks of chronic degenerative diseases [8]. In fact vegetables and fruits consist of antioxidant polyphenolic compounds, in particular flavonoids and phenolic acids, which are able to scavenge free radicals of oxygen [2,6]. Flavonoids consist of several phenolic hydroxyl functions which are subjected to glycosylation; position in the ring and numbers influence their reactivity [3,5,9].

Onion (*Allium cepa* L.) is one of the most important vegetable sources of polyphenols owing to its high flavonoids content [10]. Among onion flavonoids the quercetin and its glucosides represent 70% of total flavonoids in onions [10]. Other flavonoids present in low quantity are kaempferol, rutin, and their derivatives [7,8,9,11,12]. Since a number of polyphenols are present in onion extracts, it is important to know which ones are the major contributors to the total antioxidant activity of onions. Therefore, an extensive fractionation strategy using flash chromatography was adopted. This approach is also useful for enrichment of antioxidant polyphenol fraction which could be used for food and pharmaceutical applications. 

To characterize the antioxidant activity of plant food extracts, two different assays such as radical scavenging assay (DPPH) and ferric-reducing (FRAP) were performed on the extracts in order to complement and further ascertain the antioxidant activities [13] of the various fractions. The antioxidant capacity data were compared with total phenol and total flavonoid content data and their relationships were described.

## 2. Materials and Methods

### 2.1. Samples and Reagents

Two onion varieties (*Allium cepa* L.), i.e., red onion and yellow onion, were purchased from a local market. Onions were chopped, put in a blast freezer and subsequently freeze dried. The freeze-dried onions were grounded to a fine powder, vacuum packed, and stored at −20 °C. Methanol, ethanol, acetonitrile, formic acid, high performance liquid chromatography (HPLC)-grade water, Folin-Ciocalteu reagent, sodium carbonate (Na_2_CO_3_), gallic acid, sodium acetate anhydrous (CH_3_COONa), 37% hydrochloric acid (HCl), 6-hydroxy-2,5,7,8-tetramethylchroman-2-carboxylic acid (Trolox), Iron (III) chloride hexahydrate (FeCl_3_·6H_2_O), 2,4,6-Tripyridyl-s-Triazine (TPTZ), 2,2-Diphenyl-1-picrylhydrazyl (DPPH), aluminium chloride (AlCl_3_), potassium acetate (CH_3_COOK), quercetin, were purchased from Merck (previously Sigma-Aldrich), Wicklow, Ireland. Quercetin-4′-glucoside, quercetin-3,4′-diglucoside, cyanidin-3-glucoside and protocatechuic acid were purchased from Extrasynthese (Genay Cedex, FRANCE) and Bondesil C18 powder (40 µm) was purchased from Varian (now acquired by Agilent Technologies, Santa Clara, CA, USA).

### 2.2. Extraction of Polyphenols

In order to determine the effect of extraction time and solvent composition, approximately 1 g of powdered onion sample was mixed in 20 mL of 80% methanol and then the mixture was shaken overnight at 1500 rpm. The mixture was centrifuged for 10 min at 3000× *g*. Following this, the mixture was filtrated, and the extract was kept in the fridge at 4 °C. Aliquots of the filtrates were used to determine total phenol content, total flavonoid content, and antioxidant activities using FRAP and DPPH assays as described below.

### 2.3. Red Onion Fractionation by Flash Chromatography

**Figure 1:**
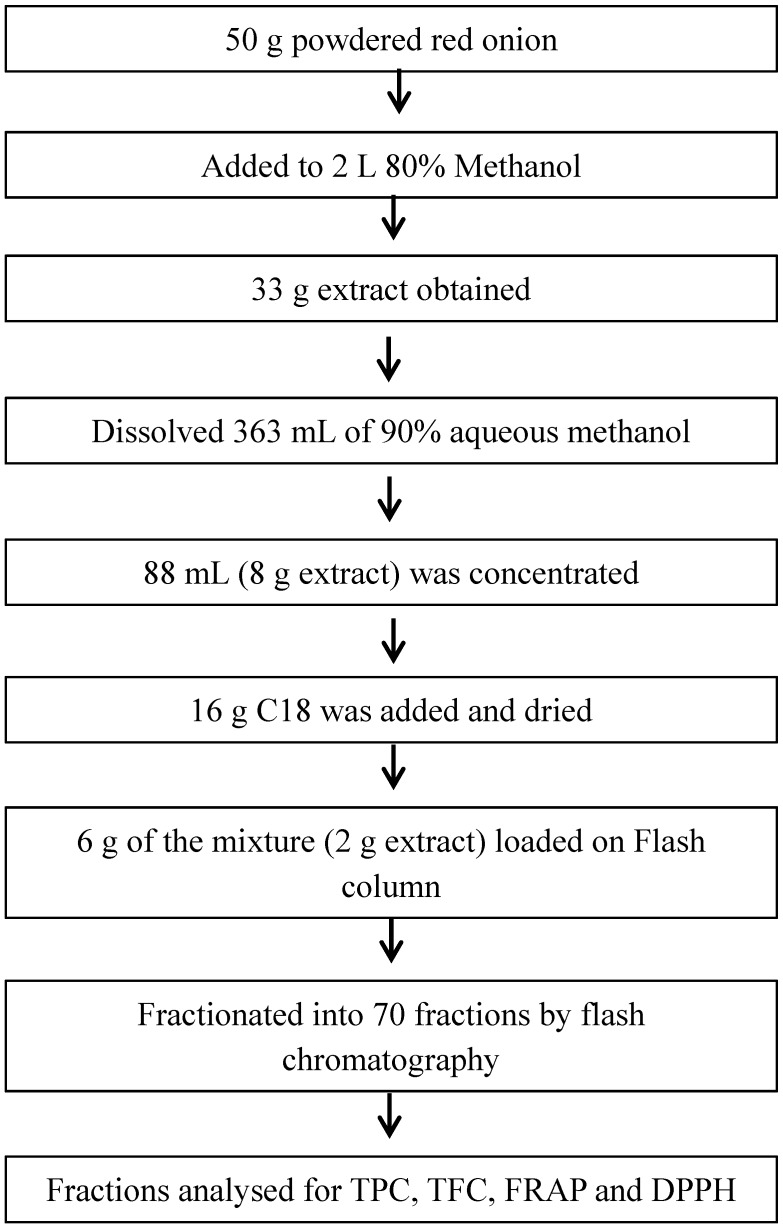
Schematic diagram of the fractionation of the onion extracts by flash chromatography.

For the flash chromatography fractionation, 50 g of powdered red onion in 1 L of 80% methanol was prepared as described in Section 2.2 with an overnight shaking, and filtrated with 0.45 μm Whatman^TM^ paper filter (GE Healthcare Life Sciences, Buckinghamshire, UK). The filtrate was put in a round-bottom flask to be dried by a rotary evaporator at 50 °C. The water in the tubes was 10.39%, which means the yield extract was over-estimated and the actual yield extract was 61.53%. To continue the sample preparation, 33 g of the semi-dried extract was diluted with 363 mL of 90% aqueous methanol; the mixture was put in the sonicator for few minutes for dissolution. An aliquot of 88 mL of this solution (8 g semi-dried extract) was concentrated in the rotary evaporator at 50 °C and then 16 g of C18 sorbent powder were added to the mixture. The mixture was dried in the rotary evaporator to remove the solvent and an aliquot of 6 g of this mixture containing 2 g of red onion extract, were placed in a loading cartridge to be fractionated by the flash chromatography (IntelliFlash 310 System, Varian, now acquired by Agilent Technologies, Santa Clara, CA, USA). A reversed phase chromatography system with a mobile phase A as water and a mobile phase B as 0.5% formic acid in acetonitrile at a flow rate of 40 mL/min was used. The flash chromatography was performed with a Telos C18 column (140 g). A stepwise gradient from 0 to 100% B in 35 min was used to separate the polyphenols and 70 fractions were recovered in a time scale of 0.5 min/fractions (Figure 1).

The UV absorptions were monitored at the wavelengths 280, 320, and 360 nm. The total phenolic and flavonoid content of all the fractions were analysed following the same methods explained below (Section 2.4 and Section 2.7), and also the antioxidant activities of these fractions were determined by DPPH and FRAP assays (Section 2.5 and Section 2.6). The highest four antioxidant fractions were chosen for the identification and quantification of the major polyphenols by UHPLC-MS/MS method (Section 2.8).

### 2.4. Determination of Total Phenol Content (TPC)

The total phenolic content of onion extracts was determined following the method of Singelton et al. [14] using Folin-Ciocalteu Reagent (FCR). Methanolic gallic acid solutions (10–400 mg/L) were used to develop a standard calibration curve. An aliquot of appropriately diluted 100 µL of the sample extract was added with 100 µL methanol, 100 µL FCR, and 700 µL Na_2_CO_3_ (20%). The mixture was vortexed and incubated for 20 min in the dark at room temperature. The mixture was centrifuged at 13,000 rpm for 3 min after incubation. The absorbance of the supernatant was measured at 735 nm by UV-Vis spectrophotometer (Hitachi U-2900, Hitachi High-Technologies Corporation, Tokyo, Japan). The experiment included two batches with three replications of each sample and standard in each batch. The total phenolic content was expressed as µg gallic acid equivalent (GAE)/mL of the extract for flash fractions and µg Trolox/g dry weight (DW) of the onion powder determining the effect of time and extraction solvent.

### 2.5. Ferric Ion Reducing Antioxidant Power (FRAP) Assay

The FRAP assay was carried out as described by Stratil et al. [15] with slight modifications. A freshly prepared FRAP reagent containing 38 mM sodium acetate anhydrous in distilled water pH 3.6, 20 mM FeCl_3_·6H_2_O in distilled water and 10 mM TPTZ in 40 mM HCl in a proportion of 10:1:1 was used. An aliquot of 100 µL of appropriately diluted sample extract was mixed with 900 µL of FRAP reagent and incubated at 37 °C for 40 min in the dark. Only methanol was used in place of sample extract in the case of the blank. The absorbance of the solution post incubation was measured by spectrophotometer (Hitachi U-2900, Hitachi High-Technologies Corporation, Tokyo, Japan) at 593 nm. Trolox, a synthetic antioxidant, at concentrations from 0.1 mM to 0.4 mM was used as a reference antioxidant standard. FRAP values were expressed as µg Trolox/mL of the extract for flash fractions and µg Trolox/g DW of the onion powder determining the effect of time and extraction solvent.

### 2.6. Determination of Free Radical Scavenging Activity by DPPH Method

DPPH assay was carried out using a modified version of the method described by Goupy et al. [16] with Trolox as a standard. Briefly, an aliquot of 500 µL of the extract or Trolox were incubated with 500 µL of a methanol solution of DPPH (0.0476 mg/mL) at room temperature for 30 min in the dark. The mixture was vortexed prior to incubation. A UV-Vis spectrophotometer (Hitachi U-2900, Hitachi High-Technologies Corporation, Tokyo, Japan) was used to measure the absorbance of the reaction mixture at 515 nm against the blank (methanol). The DPPH activity was expressed as µg Trolox/mL of the extract for flash fractions and µg Trolox/g DW of the onion powder determining the effect of time and extraction solvent.

### 2.7. Determination of Total Flavonoid Content (TFC)

Total flavonoid content was determined using the method described by Lin and Tang [17]. Briefly, the reaction mixture contained 100 μL of methanolic extract, 300 μL of 95% ethanol, 40 μL of 10% aluminium chloride, 40 μL of 1.0 M potassium acetate, and 520 μL of distilled water. The absorbance of the reaction mixture was measured against blank at 415 nm using spectrophotometer (Shimadzu UV-1700, Shimadzu Corporation, Kyoto, Japan) following an incubation period of 40 min at room temperature. Quercetin was used to develop a standard calibration curve and the total flavonoid content was expressed as µg Quercetin equivalent (QE)/mL of the extract for flash fractions and µg Trolox/g DW of the onion powder determining the effect of time and extraction solvent.

### 2.8. Characterisation and Quantification by UHPLC-MS/MS

The polyphenols in the selected fractions were identified and quantified using Waters Acquity (Waters Corporation, Milford, MA, USA) ultra-high performance liquid chromatography coupled with tandem quadrupole mass spectrometry (UHPLC-TQD-MS). The LC separation of the analytes was performed on a Waters Acquity HSS T3 UHPLC column (1.8 μm, 2.1 × 100 mm) using milli-Q^®^ (18 mΩ) (Merck Millipore, Molsheim, France) water (mobile phase A) and acetonitrile:methanol (1:1) containing 0.5% formic acid (mobile phase B). A gradient program of 0–2.5 min 2% B, 2.5–3 min 10% B, 3–7.5 min 15% B, 7.5–8.5 min 35% B, 8.5–9.5 min 98% B, and 9.5–10.0 min 2% B at a flow rate of 0.5 mL/min was used. A multiple reaction monitoring (MRM) approach was taken for the mass spectrometric determination of the polyphenols using argon as collision gas. The parameters for MRM transitions were obtained using the Waters integrated Intellistart^TM^ software (Waters Corp., Milford, MA, USA) (Table 1).

The UHPLC-MS/MS data were acquired using electrospray ionisation in negative ion mode (ESI–) with the following ionisation conditions: capillary voltage 3 kV, cone voltage 42 V, extractor voltage 3 V, source temperature 150 °C, desolvation gas flow 1200 L/h, and desolvation temperature 350 °C.

## 3. Results and Discussion

**Figure 2:**
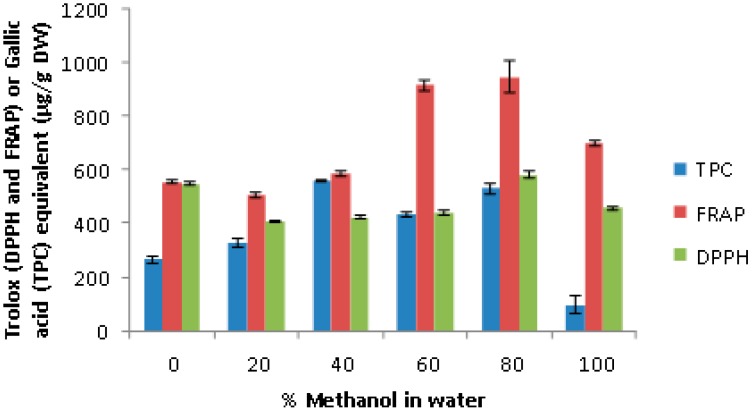
Effect of different methanolic compositions on the antioxidant activity (2,2-diphenyl-1-picrylhydrazyl (DPPH) and ferric reducing antioxidant power (FRAP)) and total phenol content (TPC) of the yellow onion extract.

A number of studies have shown aqueous-alcohols particularly 80% methanol to be highly efficient in extracting antioxidant polyphenols from plant matrices [1,9]. However, samples having different polyphenol profiles might respond differently to various extracting solvent compositions. Therefore, a range of methanolic concentrations in water (0–100%) for their efficacies to extract antioxidant polyphenols was tested, where the 80% methanol as expected from the previous studies has shown the highest total phenol content and antioxidant capacity (Figure 2).

**Figure 3:**
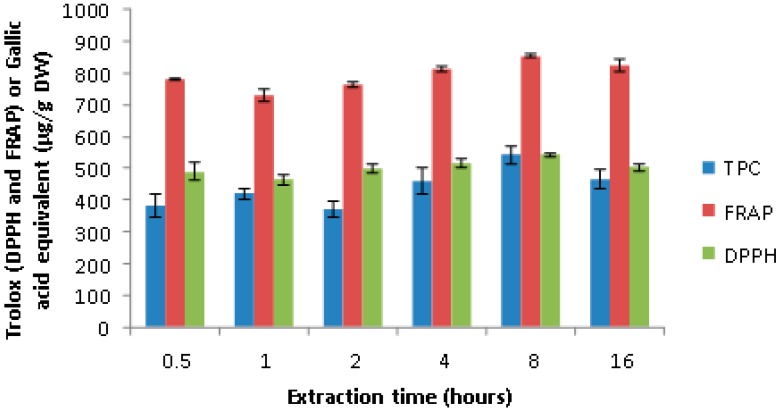
Effect of time of extraction on the antioxidant activity and total phenol content of yellow onion extract.

In addition, the best extraction time was determined by testing the extracts for total phenol content and antioxidant capacity for the extracts obtained at 0.5, 1, 2, 4, 8, and 16 h of extraction duration. The results suggested that the extracts obtained at 8 h interval had the highest total phenol content and antioxidant capacity among the time intervals tested (Figure 3). This indicated that the extraction of antioxidant polyphenols reached its maximum at an eight hour interval and there was a slight degradation during prolonged extraction for 16 h. 

Preliminary data on antioxidant activities of red and yellow onions showed that red onions had significantly higher values than those of yellow onions. For this reason, in the subsequent fractionation experiment where a larger volume of extract was carried out using 80% methanol for eight hours using freeze-dried red onion powder. The total phenol contents of onion extracts were in the range 520 ± 10 µg GAE/g DW to 13370 ± 80 µg GAE/g DW. These results were in agreement with the data presented by Ren et al. [18]. The total flavonoid showed the similar results to Ren et al. ranging from 340 ± 10 µg QE/g DW for yellow onion to 7130 ± 170 µg QE/g DW for red onion. Moreover we observed that the TFC represented 53.3% of TPC in red onion while it reached 83.2% in yellow onion. In line with the TPC and TFC values, the antioxidant activity, analysed by DPPH and FRAP assays, was higher in the red variety. A good correlation between TPC and FRAP (r^2^ = 0.9663) and between TPC and DPPH (r^2^ = 0.8126) was observed. The total phenols assay by Folin-Ciocalteu reagent allows measuring the reducing capacity of the sample and many results show a linear correlation between antioxidant capacity and phenol content. Indeed, in most cases, antioxidant responses of plant extracts are in agreement with the content of their polyphenol compounds [4,9]. The FRAP assay allows estimating the reducing capacity of the sample whilst the DPPH assay measures radical scavenging activities [4]. The FRAP assay showed a better sensitivity for all the extracts and it is well correlated with DPPH radical scavenging data (r^2^ = 0.8464). In onions, the present study suggests that whatever the varieties, phenolic compounds are one of the principal antioxidant compounds. 

**Figure 4:**
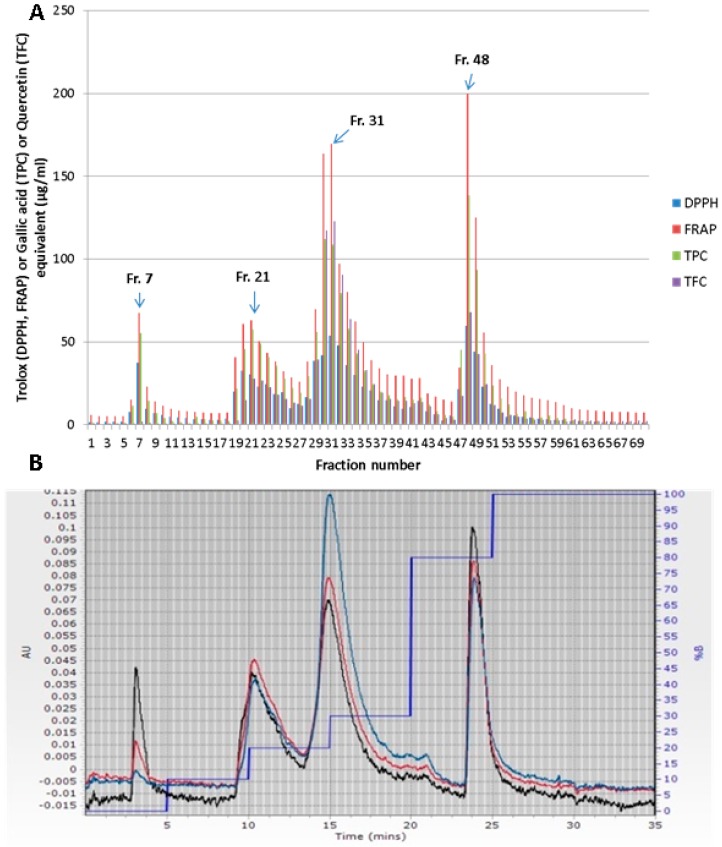
(**A**) Antioxidant activities, total phenol, and total flavonoid contents of the fractions obtained from flash chromatography of red onion extract, (**B**) Flash chromatogram of the crude red onion extract (blue, red, and black lines indicated the chromatograms obtained at 360 nm, 320 nm, and 280 nm wavelengths respectively) Fraction numbers in 3A equals time intervals in 3B.

Crude red onion extract was partitioned into 70 fractions during a run time of 35 min using flash chromatography to separate the compounds, mainly based on polarity, on C18 flash column. These 70 fractions were screened for their total phenol content (TPC), total flavonoid content (TFC), and antioxidant activity as measured by DPPH and FRAP assays (Figure 4A). The flash separation of the crude onion extract had produced four major peaks. The graph of antioxidant activities of the fractions resembled the flash chromatogram showing a good correlation among the constituents of the peaks, predominantly phenolic compounds and antioxidant activities (Figure 4A,B). Among the fractions, fraction number 48 had the highest total phenol content and antioxidant capacity followed by fraction 31. This shows the major polyphenols in onion were of intermediate polarity. Moderately polar fraction 21 and highly polar fraction seven had relatively low TPC and antioxidant capacity. These fractions were representatives of the four main peaks observed in flash chromatography. Therefore, these fractions were further investigated for their constituent polyphenols and a link between their polyphenol profile and antioxidant activity was observed.

The main constituent polyphenol in fraction 48 was quercetin which constituted approximately 95% of the total ion count of the peak while less intense peaks of quercetin-4′-glucoside and quercetin-3,4′-diglucoside were also observed (Table 2). On the other hand, these glycosidic quercetins were the main polyphenols in fractions 31 and 21 with quercetin-3,4′-diglucoside eluting earlier mainly in fraction 21 having an ion count of 3.2 × 10^5^. These two fractions also contained considerably intense peak of cyanidin-3-glucoside with ion counts of 3.1 × 10^5^ and 1.2 × 10^5^, respectively (Table 2). 

The highly polar fraction 7 contained mainly protocatechuic acid and trace amount of quercetin-3,4′-diglucoside. Antioxidant capacity test of these individual polyphenols has revealed that quercetin had the highest antioxidant capacity among the flavonoids (Table 3). This could be explained by the fact that it had all the structural features of a flavonoid relevant to its antioxidant potential [19]. The antioxidant capacity of the quercetin aglycones had higher antioxidant capacity than their glycosidic counterparts. Glycosylation of the free hydroxyl groups of polyphenols generally reduces their antioxidant potential [19]. This was reflected in the high antioxidant capacity of fraction 48. Although the protocatechuic acid of fraction 7 had considerably high antioxidant capacity (Table 3), presence of this phenolic acid in fraction 7 in low quantity accounted for the low antioxidant capacity of fraction 7.

This finding suggests that antioxidant capacity of a fraction is a combined function of both quantity and antioxidative reactive potential of the constituent compounds. This was further confirmed when we analysed the antioxidant activity and quantity of total flavonoid of the fractions 31 and 48. Fraction 48 had lower total flavonoid content than fraction 31 while it had the highest antioxidant activity due to the presence of quercetin which was the strongest antioxidant among onion flavonoids. In this case lower quantity was compensated by high activity. From the chromatographic separation point of view, the peak at 15 min was wider than the peak at 24 min. Therefore, the peak at 15 min had more fractions (fraction 29 to 36) than those of the peak at 24 min (fraction 47 to 52). The combined antioxidant capacities of all the fractions of the peak at 15 min surpassed the combined antioxidant capacities of the fractions that belonged to peak at 24 min. In fact, the combined antioxidant capacities of the fractions of peak at 15 min constituted 33.4% and 33.3% of the total antioxidant capacity as measured by DPPH and FRAP methods, respectively. Whereas, the fractions of peak at 24 min contributed 18.5% and 20.6% of the total DPPH and FRAP values of the crude extract. This result suggested that the quercetin glycosides are the main contributor to antioxidant capacity of onion extract although aglycone quercetin had higher antioxidant capacity than that of the quercetin glycosides. The predominant anthocyanin of onion extract cyanidine-3-glucoside also contributed considerably to the total antioxidant capacity of the fractions around 10 and 15 min. Flash chromatographic separation of the crude extract was very useful for the separation and enrichment of the antioxidant polyphenols. It has been observed that approximately 90% of the crude onion extract was non-phenolic and non-antioxidant compounds which eluted at early stage of the chromatographic run. Although a small amount of protocatechuic acid was eluted with these highly polar non-antioxidant compounds, this phenolic acid constituted only 7.5% of total DPPH and 5.6% of total FRAP values of the crude extract. Therefore, by removing these non-antioxidant compounds from the crude extracts, an enrichment of >9 folds in antioxidant capacity of the remaining extract was achievable. This removal left the fractions from the second peak and onward (fraction 21 to 70) predominantly composed of polyphenols. Moreover, the fractions at the centre of the peaks obviously had higher enrichment of single polyphenol. This one step easy to perform flash chromatographic enrichment approach of quercetin and its derivatives is highly relevant for food and phytopharmaceutical industries which are currently seeking naturally occurring highly active antioxidant compounds.

## 4. Conclusions

Flash chromatographic fractionation technique was a useful tool to isolate and enrich major polyphenols in red onion extract. Subsequent antioxidant activity and mass spectrometric analyses identified and quantified main antioxidant polyphenols in flash fractions. Flavoniods, quercetin glycosides in particular were the dominant antioxidant polyphenols of red onion extract. The total antioxidant capacity of a fraction was a function of of both quantity and antioxidative reactive potential of the constituent compounds.

## Figures and Tables

**Table 1:** MRM transitions, cone voltage and collision energy for determination of the polyphenols.

Standards	Molecular Formula	Retention Time (min)	MRM (*m*/*z*)	Quantifier Ion (*m*/*z*)	Cone Voltage (V)	Collision Energy (eV)
Protocatechuic acid	C_7_H_6_O_4_	1.55	152.9→80.9	108.9	29	18
			→108.9			14
Cyanidin-3-glucoside	C_21_H_21_O_11_	2.77	447.2→285.2	285.2	57	32
			→269.7			22
Quercetin-3,4′-diglucoside	C_27_H_30_O_17_	3.36	625.1→300.0	463.0	57	42
			→463.0			18
Quercetin-4′-glucoside	C_21_H_20_O_12_	4.71	463.0→300.0	300.0	52	26
Quercetin	C_15_H_10_O_7_	6.89	301.0→106.9	150.9	33	28
			→150.9			24
			→178.9			18

**Table 2:** Ion counts and quantity of the antioxidant compounds of the main fractions of red onion extract.

Fraction Number	Main Compound	Ion Count	Quantity (mg/fraction)
7	Protocatechuic acid	1.8 × 10^5^	3.05
	Quercetin-3,4′-diglucoside	0.5 × 10^3^	0.14
21	Quercetin-3,4′-diglucoside	3.2 × 10^5^	6.19
	Quercetin-4′-glucoside	1.2 × 10^4^	0.35
	Cyanidin-3-glucoside	3.1 × 10^5^	3.30
31	Quercetin-3,4′-diglucoside	3.1 × 10^5^	6.00
	Quercetin-4′-glucoside	3.2 × 10^5^	6.81
	Cyanidin-3-glucoside	1.2 × 10^5^	1.42
48	Quercetin	2.08 × 10^5^	2.29

**Table 3:** Antioxidant capacity of the main compounds of red onion extract.

Compound Name	Antioxidant Activity	
	DPPH (µg Trolox/100 µg DW)	FRAP (µg Trolox/100 µg DW)
Protocatechuic acid	25.61 ± 2.65	48.63 ± 4.28
Cyanidine-3-glucoside	21.32 ± 2.02	44.75 ± 3.05
Quercetin-3,4′-diglucoside	20.24 ± 3.20	42.05 ± 3.21
Quercetin-4′-glucoside	27.10 ± 1.59	51.03 ± 3.51
Quercetin	32.56 ± 2.64	60.19 ± 3.62
